# Report on planning comparison of VMAT, IMRT and helical tomotherapy for the ESCALOX-trial pre-study

**DOI:** 10.1186/s13014-020-01693-2

**Published:** 2020-11-02

**Authors:** Steffi U. Pigorsch, Severin Kampfer, Markus Oechsner, Michael C. Mayinger, Petra Mozes, Michal Devecka, Kerstin K. Kessel, Stephanie E. Combs, Jan J. Wilkens

**Affiliations:** 1 Department of Radiation Oncology, Technical University of Munich (TUM), School of Medicine, Klinikum Rechts Der Isar, Ismaninger Straße 22, 81675 Munich, Germany; 2 Institute of Radiation Medicine (IRM), Helmholtz Zentrum München, Ingolstädter Landstraße 1, Neuherberg, Germany; 3 Department of Radiation Oncology, University Hospital Zurich, Rämistrasse 100, Zurich, Switzerland

**Keywords:** Dose escalation, Head and neck cancer, Combined chemoradiation therapy, RT planning comparison, IMRT, VMAT, Helical tomotherapy, SIB

## Abstract

**Background:**

The ESCALOX trial was designed as a multicenter, randomized prospective dose escalation study for head and neck cancer. Therefore, feasibility of treatment planning via different treatment planning systems (TPS) and radiotherapy (RT) techniques is essential. We hypothesized the comparability of dose distributions for simultaneous integrated boost (SIB) volumes respecting the constraints by different TPS and RT techniques.

**Methods:**

CT data sets of the first six patients (all male, mean age: 61.3 years) of the pre-study (up to 77 Gy) were used for comparison of IMRT, VMAT, and helical tomotherapy (HT). Oropharynx was the primary tumor location. Normalization of the three step SIB (77 Gy, 70 Gy, 56 Gy) was D95% = 77 Gy. Coverage (CVF), healthy tissue conformity index (HTCI), conformation number (CN), and dose homogeneity (HI) were compared for PTVs and conformation index (COIN) for parotids.

**Results:**

All RT techniques achieved good coverage. For SIB77Gy, CVF was best for IMRT and VMAT, HT achieved highest CN followed by VMAT and IMRT. HT reached good HTCI value, and HI compared to both other techniques. For SIB70Gy, CVF was best by IMRT. HTCI favored HT, consequently CN as well. HI was slightly better for HT. For SIB56Gy, CVF resulted comparably. Conformity favors VMAT as seen by HTCI and CN. Dmean of ipsilateral and contralateral parotids favor HT.

**Conclusion:**

Different TPS for dose escalation reliably achieved high plan quality. Despite the very good results of HT planning for coverage, conformity, and homogeneity, the TPS also achieved acceptable results for IMRT and VMAT.

*Trial registration* ClinicalTrials.gov Identifier: NCT 01212354, EudraCT-No.: 2010-021139-15. ARO: ARO 14-01

## Background

Head and neck cancer patients are treated by intensity modulated radiotherapy (IMRT) as standard of care in radiation oncology. Early in the era of IMRT application, the concept of the simultaneous integrated boost (SIB) was evaluated [1]. This boost technique creates a selective heterogeneous dose distribution in one target divided into subvolumes with the aim of better conformity [2]. Many results of planning comparisons using different treatment planning systems (TPS) and radiotherapy (RT) treatment concepts have been published for both head and neck as well as other cancer. Many of these used real patient data sets for comparison of treatment modalities at the same institution [3–9]. IMRT, volumetric modulated arc therapy (VMAT), and helical tomotherapy (HT) are routinely used to treat patients with head and neck cancer.

In multicenter prospective trials with IMRT, quality assurance (QA) of RT planning is mandatory [10, 11]. QA for RT of head and neck cancer does not only include the quality of RT plans, but begins earlier in the treatment process. The delineation of the target and organ at risk (OAR) requires standardization and quality control. Especially with the implementation of RT plans with steep dose gradients, the correct target volume delineation is of outstanding importance. Otherwise the clinical outcome of head and neck cancer patients is compromised [11]. Feasibility of the RT-strategy is the first step of RT-trial design. The second step is the proof of dose specification with different TPS, followed by a dummy run of all participating trial centers including a dummy run for target volume and OAR contouring.

The ESCALOX trial was planned as a multicenter trial for dose escalation in head and neck cancer to the gross tumor volume (GTV) defined by CT and MRI imaging in 2010. FMISO-PET was part of a translational research project and not implemented in target volume definition. Data sets of the first six patients of the pre-study as determined by the German Federal Office of Radiation Protection (Bundesamt für Strahlenschutz—BfS) (BfS-registration number Z5-22463/2-2011-011) were used for planning comparison with different TPS. The aim of this planning study was to compare the non-uniform dose prescription and calculated planning target volume (PTV) doses of the different SIB volumes as well as the calculated doses to the OARs between two TPS: Eclipse version 13.0 (Varian Medical Systems, Palo Alto, CA, USA) for VMAT (RapidArc) and dynamic multi-field IMRT, and TomoTherapy Planning Station version 4.2.3 (Accuray, Sunnyvale, CA, USA).

The hypothesis of this investigation was the generation of comparable RT plans via different TPS and RT techniques respecting the dose prescription to the SIB volumes as a primary objective. The secondary planning goal was the concurrent minimization of dose to the organs at risk (OAR) while having an escalated dose of up to 77 Gy in the SIB.

## Methods

### Patient characteristics and RT concept

Patients were treated between 1/2016 and 2/2017 after inclusion in the pre-study of the ESCALOX trial at the Department of Radiation Oncology of the Technical University of Munich. All included patients were male with a smoking history of more than 10 packyears. The mean age was 61.3 years [53–70 years]. In all cases, primary tumor location was the oropharynx, for details, see Table 1. TNM classification was done according to the 7th edition of UICC, 2010. The mean GTV of primary tumor was 62.6 cm^3^ [IQR 22.4–116.4 cm^3^]. Concomitant platin-based chemotherapy was standard of care in all patients. All patients were treated with 35 fractions with 5 fractions per week. Because of the three step SIB concept, each target volume was deemed as SIB: SIB77Gy: 2.2 Gy up to 77 Gy; SIB70Gy: 2.0 Gy up to 70 Gy and SIB56Gy: 1.6 Gy up to 56 Gy (for details see [12]).

**Table 1:** Patient characteristics

Characteristics	GTV = SIB77Gy (cm^3^)
Patient 1, male Age at diagnosis: 67 years Diagnosis: OPC (midline of base of tongue) TNM: cT3 cN2b cM0 G3 Histology: SCC, p16+ Treatment: CCRT once Cisplatin 40 mg/m^2^/day, change to 5× Carboplatin AUC 2 (only one kidney after accident)	86.8
Patient 2, male Age at diagnosis: 70 years Diagnosis: OPC (base of tongue right) TNM: cT2 cN2c cM0 G3 Histology: SCC, p16− Treatment: CCRT with 4× Cisplatin 40 mg/m^2^/day	77.33
Patient 3, male Age at diagnosis: 53 years Diagnosis: OPC (left soft palate, base of tongue, tongue left side, floor of mouth, exceeding midline) TNM: cT4a cN2c cM0 G3 Histology: SCC, p16− Treatment: CCRT with 6× Cisplatin 40 mg/m^2^/day	116.4
Patient 4, male Age at diagnosis: 54 years Diagnosis: OPC (left tonsil, submandibular gland, dorsal third of tongue, fossa pterygopalatina) TNM: cT4a cN2c cM0 G3 Histology: SCC, p16− Treatment: CCRT with 6× Cisplatin 40 mg/m^2^/day	24.6
Patient 5, male Age at diagnosis: 56 years Diagnosis: OPC (left tonsil and fossa) TNM: cT2 cN2b cM0 G2 Histology: SCC, p16− Treatment: CCRT with 6× Cisplatin 40 mg/m^2^/day	48.3
Patient 6, male Age at diagnosis: 68 years Diagnosis: OPC (right tonsil and soft palate) TNM: cT4a cN2b cM0 G3 Histology: SCC, p16− Treatment: CCRT with 3× Cisplatin 40 mg/m^2^/day	22.4

### Target delineation

Head and shoulder mask systems were customized before treatment planning CT for all patients (slice thickness 3 mm, intravenous contrast enhancement in all cases). CT extension comprised base of skull to mid-mediastinum. The CT images were reconstructed with 512 × 512 pixel matrices.

Before treatment planning, every patient was also staged by means of a head and neck MRI. These MRI scans were co-registered to the planning CT. Based on the panendoscopy report and the imaging entirety, the GTV, clinical target volume (CTV), and SIB (PTV) were delineated and crosschecked by two radiooncologists (MD, SP). Bilateral neck lymph drainage was PTV in all cases. Every isotropic margin generation by the TPS was re-contoured manually with respect to anatomic structures (bones, ligaments, cartilage, muscle) usually not tumor-invaded as recommended by trial protocol. The RT plans were evaluated quantitatively and qualitatively (isodose curves and color wash) by two physicians.

### Treatment planning—VMAT, IMRT, HT

All plans were calculated, optimized, and approved by experienced medical physicists on the particular system (Eclipse for VMAT and IMRT, and TomoTherapy Planning Station for HT). Normalization was done to 95% of the volume with the highest prescribed dose (SIB77). In the tomotherapy planning workflow, normalization is done during the optimization process. Hence, there can be small discrepancies between the “normalization dose” and the dose after final dose calculation (after which the dose will not be normalized again). The first optimization goal was to bring 95% of the PTV to the specific prescribed doses (Table 2). In addition, 50% of the PTV volumes (SIB77Gy, SIB70Gy, SIB56Gy) were to receive no more than 79.3 Gy, 72.1 Gy, and 57.7 Gy, respectively. The second planning objective was to spare the following OARs according to their constraints or to the ALARA principle: spinal cord (spinal cord + 5 mm) (mandatory), brachial plexus (mandatory), brainstem (mandatory), optical nerve (mandatory), mandible, glottis, and parotid glands. For OARs labeled as “mandatory”, the given dose was a hard constraint. In some cases, when it was not possible to fulfill both criteria (PTV dose and mandatory OAR dose—see Table 3), a lower dose in the PTV was accepted (detailed delineation and planning are described in [12]). All planning modalities used identical dose criteria for the SIBs and OARs as described in the trial protocol.

**Table 2:** ESCALOX—trial dose prescription for SIB-Volumes volumes

PTV (SIB)	Planning goal D_95%_ (Gy)	Planning goal D_50%_ (Gy)
SIB77Gy	77	≤ 79.3
SIB70Gy	70	≤ 72.1
SIB56Gy	56	≤ 57.7

**Table 3:** Dose constraints for OAR defined by the ESCALOX trial protocol

OAR	D_95%_	Planning goals as accepted dose [EQD2]
Spinal cord (PRV: spinal cord + 5 mm)	Mandatory	D_max_ < 50 Gy_2_ or No more than 1 cm^3^ > 45 Gy_2_
Brachial plexus	Mandatory	60 Gy_2_
Brainstem	Mandatory	D_max_ < 54 Gy_2_ or No more than 1 cm^3^ > 54 Gy_2_
Optical nerve	Mandatory	D_max_ < 54 Gy_2_
Mandible	Recommended	D_max_ < 70 Gy_2_ or No more than 1 cm^3^ > 70 Gy_2_
Glottis (outside PTV)	Recommended	2/3 < 50 Gy_2_ D_mean_ < 45 Gy_2_
Gl. parotis	Recommended	D_mean_ < 26 Gy_2_ D_50_ < 30 Gy_2_ 20 cm^3^ of both < 20 Gy_2_

#### VMAT and IMRT

Treatment planning for the VMAT and dynamic IMRT plans was performed with the Eclipse 13.0 TPS. For dose calculation, the Anisotropic Analytical Algorithm (AAA, version 10.028) was used with a dose grid size of 2.5 × 2.5 × 3.0 mm^3^. VMAT and IMRT planning was performed for treatment using a Varian Clinac Trilogy linear accelerator equipped with a 120 HD MLC. VMAT plans consisted of three arcs with 358° rotation each. The sliding window IMRT plans were optimized using a beam set-up of nine coplanar static beam directions with evenly distributed angular distances.

All plans were normalized to a dose of 77 Gy, covering 95% of the SIB77Gy volume as described in the trial protocol [12]. There were no limits for treatment planning time. Re-optimization for treatment planning was allowed and recommended to push the TPS to the limits.

#### Helical tomotherapy

Treatment planning was done with the TomoTherapy Planning Station (Accuray), version 4.2.3. The tomotherapy plans were all helical IMRT plans with field widths of 1 cm and calculation grid size set to fine.

### Dose-volume histogram (DVH) analysis and statistics

All DVH analyses were done using the Eclipse TPS (HT plans were imported). DVH data was extracted to a TXT-file and then read out by R-Software (R version 3.4.1 (2017-06-30)—The R Foundation for Statistical Computing) and spreadsheets. Statistical analysis was calculated by IBM SPSS version 25. The DVH data of each patient was compared for every planning approach (IMRT, VMAT or HT) and between all patients by calculating the mean data for every parameter.

A comparison of the mean DVH data of all techniques was made by using Student’s two-sided paired t-test or Wilcoxon test, depending on the distribution of the values (IMRT vs. HT; IMRT vs. VMAT and HT vs. VMAT). Statistical significance was supposed for p ≤ 0.05.

### Parameters for DVH analysis 

To compare different RT-plans (VMAT, IMRT and HT), the following DVH-SIB (PTV) parameters were rated: SIB77Gy—D_mean_, D_100%_, D_98%_ (D_min_), D_25%_, D_5%_ and D_2%_ (D_max_). We defined the SIB70Gy-volume as a shell without the inside-located SIB77Gy-volume. For the SIB56Gy-volume, the same procedure was done to create the shell without SIB70Gy- and SIB77Gy-volumes. For each shell (SIB56Gy and SIB70Gy), D_95%_ and D_5%_ are reported (Table 4) (for raw data see Additional file 1). For assessment of plan quality, the coverage factor (CVF), healthy tissue conformity index (HTCI), conformation number (CN) and the homogeneity index (HI) were calculated and compared to each other [3, 13]. These parameters were used to judge the quality of the different planning target volumes (i.e. SIB).

**Table 4:** Treatment planning results: Dose-volume histogram parameters and coverage, conformity and homogeneity indices for VMAT, IMRT, and HT (mean ± SD); ipsilateral corresponds to the tumor site, contralateral to the opposite side of tumor location

Volume	Para-meter	Unit	VMAT	IMRT	HT	p-value		
						IMRT vs. HT	IMRT vs. VMAT	HT vs. VMAT
SIB77Gy	Volume	cm^3^ (range)	54.2 ± 35.5 Median 37.7					
	D_mean_	Gy	79.1 ± 0.2	79.2 ± 0.2	78.5 ± 0.7	0.048	0.3	0.018
	D_100%_	Gy	72.9 ± 1.0	72.7 ± 0.7	73.6 ± 1.7	0.3	0.3	0.5
	D_98%_	Gy	76.3 ± 0.2	76.3 ± 0.2	76.2 ± 1.3	0.9	0.5	0.9
	D_95%_	Gy	77.0 ± 0	77.0 ± 0	77.2 ± 0.1	0.6	0.1	0.6
	D_2%_	Gy	81.6 ± 0.2	81.5 ± 0.5	79.8 ± 1.0	0.015	0.9	0.013
	D_5%_	Gy	81.2 ± 0.2	81.2 ± 0.5	79.6 ± 1.0	0.026	1.0	0.017
Coverage	CVF		1 ± 0.00	1 ± 0.00	0.997 ± 0.007			
Conformity	HTCI		0.25 ± 0.10	0.27 ± 0.11	0.37 ± 0.03			
	CN		0.25 ± 0.10	0.27 ± 0.11	0.37 ± 0.03			
Homogeneity	HI		1.054 ± 0.003	1.054 ± 0.006	1.032 ± 0.012			
SIB70Gy (excluding SIB77Gy)	Volume	cm^3^	257.4 ± 112.7 Median 220.7					
	D_95%_	Gy	69.5 ± 0.2	69.8 ± 0.3	69.1 ± 0.8	0.2	0.2	0.4
	D_5%_	Gy	77.8 ± 0.2	76.9 ± 0.6	76.7 ± 0.4	0.1	0.6	0.044
Coverage	CVF		0.995 ± 0.002	0.997 ± 0.002	0.995 ± 0.005			
Conformity	HTCI		0.759 ± 0.026	0.773 ± 0.032	0.827 ± 0.031			
	CN		0.754 ± 0.024	0.769 ± 0.032	0.823 ± 0.032			
Homogeneity	HI		1.145 ± 0.01	1.142 ± 0.008	1.141 ± 0.015			
SIB56Gy (excluding SIB70Gy)	Volume	cm^3^ (range)	691.9 ± 181.2 Median 653.5					
	D_95%_	Gy	55.4 ± 0.2	55.3 ± 0.4	55.4 ± 0.5	1.0	0.5	0.4
	D_5%_	Gy	69.3 ± 0.3	68.9 ± 0.8	67.5 ± 0.8	0.5	0.1	0.015
Coverage	CVF		0.986 ± 0.004	0.985 ± 0.004	0.983 ± 0.005			
Conformity	HTCI		0.747 ± 0.024	0.731 ± 0.027	0.728 ± 0.031			
	CN		0.739 ± 0.023	0.723 ± 0.029	0.719 ± 0.031			
Homogeneity	HI		1.391 ± 0.018	1.384 ± 0.018	1.381 ± 0.020			
Organs at risk								
Parotid Glands both	COIN		0.606 ± 0.040	0.593 ± 0.032	0.603 ± 0.065			
ipsilateral	D_mean_	Gy	32.8 ± 4.8	33.1 ± 4.4	31.1 ± 4.0	0.046	0.345	0.043
	Volume	cm^3^	30.4 ± 6.0					
	COIN		0.555 ± 0.047	0.548 ± 0.040	0.546 ± 0.073	0.917	0.345	0.753
Contralateral	D_mean_	Gy	26.3 ± 3.9	26.2 ± 3.6	22.6 ± 5.5	0.116	0.173	0.043
	Volume	cm^3^	26.9 ± 5.5					
	COIN		0.665 ± 0.043	0.647 ± 0.040	0.664 ± 0.063	0.345	0.028	0.917
Spinal cord + 5 mm	D_max_	Gy	50.5 ± 1.4	51.9 ± 1.7	45.7 ± 7.4			
Brainstem	D_max_	Gy	27.4 ± 7.45	29.07 ± 1.9	25.94 ± 5.9	0.1	0.1	0.037
Larynx	D_mean_	Gy	45.2 ± 6.1	44.7 ± 5.8	43.3 ± 8.7			
Plexus brachialis Ipsilateral	D_2%_	Gy	58.5 ± 0.4	58.0 ± 0.9	57.6 ± 1.6	0.345	0.345	0.138
	COIN		0.306 ± 0.159	0.398 ± 0.433	0.505 ± 0.172	0.116	0.075	0.028
Plexus brachialis contralateral	D_2%_	Gy	58.5 ± 0.3	58.1 ± 0.3	57.9 ± 1.4	0.463	0.046	0.138
	COIN		0.279 ± 0.141	0.355 ± 0.155	0.535 ± 0.178	0.028	0.028	0.028
Mandibula	D_mean_	Gy	46.0 ± 7.2	48.7 ± 6.1	44.2 ± 7.7	0.014	0.025	0.1
	D_2%_	Gy	70.7 ± 3.3	71.2 ± 2.6	71.9 ± 2.5	0.2	0.6	0.2

The formulae to calculate all these parameters are given with:

1. CVF—coverage factor [13] $\mathrm{CVF}=\frac{\mathrm{TVRI}}{\mathrm{TV}}$
  TVRI—irradiated target volume encompassed by reference dose 95%-isodose; TV—target volume.
2. HTCI—healthy tissue conformity index [14] $\mathrm{HTCI}=\frac{\mathrm{TVRI}}{\mathrm{VRI}}$
  VRI—irradiated volume encompassed by reference 95%-isodose.
3. CN—conformation number [15] $\mathrm{CN}=\mathrm{CVF }\times \mathrm{HTCI}$
4. HI—homogeneity index [13] $\mathrm{HI}=\frac{{\mathrm{D}}_{5\mathrm{\%}}}{{\mathrm{D}}_{95\mathrm{\%}}}$
  Parameters for OAR for head and neck cancer were reported from DVH and also compared depending on the RT technique (parallel OAR: parotid gland D_mean_ [16–18]; larynx D_mean_ [19], serial OAR: spinal cord + 5 mm D_max_; brainstem D_max_, plexus brachialis D_2%_, mandibula D_mean_ and D_2%_). In order to properly assess the OAR doses of the parotid gland, it is important to note that three patients had left-sided and two patients right-sided cancer of the oropharynx. In one case, the tumor was located near the midline with growth to the right. In this way, we used “ipsilateral” parotid gland as the site of primary tumor location and contralateral for the primary tumor free zone. In order to compare the parotid glands (ipsi- or contralateral to the primary tumor), the conformity index was used.
5. COIN—conformity index according to Baltas et al. [20]
  $$COIN=CN\times (1-\frac{{V}_{OAR ref}}{{V}_{OAR}}$$).
  V_OARref_ OAR volume receiving reference dose; V_OAR_ total volume of OAR.

## Results

All RT plans achieved good to excellent coverage (CVF) for the SIB-volumes. SIB77Gy for IMRT and VMAT had a CVF value of 1.0 and HT achieved a CVF value of 0.997 ± 0.007. HT yielded best HTCI (HT 0.372 ± 0.034 vs. VMAT 0.254 ± 0.103 vs. IMRT 0.273 ± 0.123) and CN (HT 0.371 ± 0.033 vs. VMAT 0.254 ± 0.103 vs. IMRT 0.273 ± 0.123), see Table 4.

As for coverage and conformity, HT achieved the best result for dose homogeneity (HI: HT 1.032 ± 0.013 vs. VMAT 1.054 ± 0.003 vs. IMRT 1.054 ± 0.006).

SIB70Gy had best coverage with the IMRT plan (CVF 0.997 ± 0.002 vs. 0.995 ± 0.002 for VMAT and 0.995 ± 0.005 for HT). HT achieved a very good HTCI (0.827 ± 0.034) vs. 0.759 ± 0.01 for VMAT and 0.773 ± 0.035 for IMRT. As a consequence of the high HTCI, the tomotherapy plan reached the highest conformation number (CN) 0.823 ± 0.035 compared to IMRT (CN 0.769 ± 0.035) and VMAT (CN 0.754 ± 0.024) for SIB70Gy. Homogeneity calculation favors HT (HI: HT 1.141 ± 0.016 vs. IMRT 1.142 ± 0.009 vs. VMAT 1.145 ± 0.01).

**Fig. 1:**
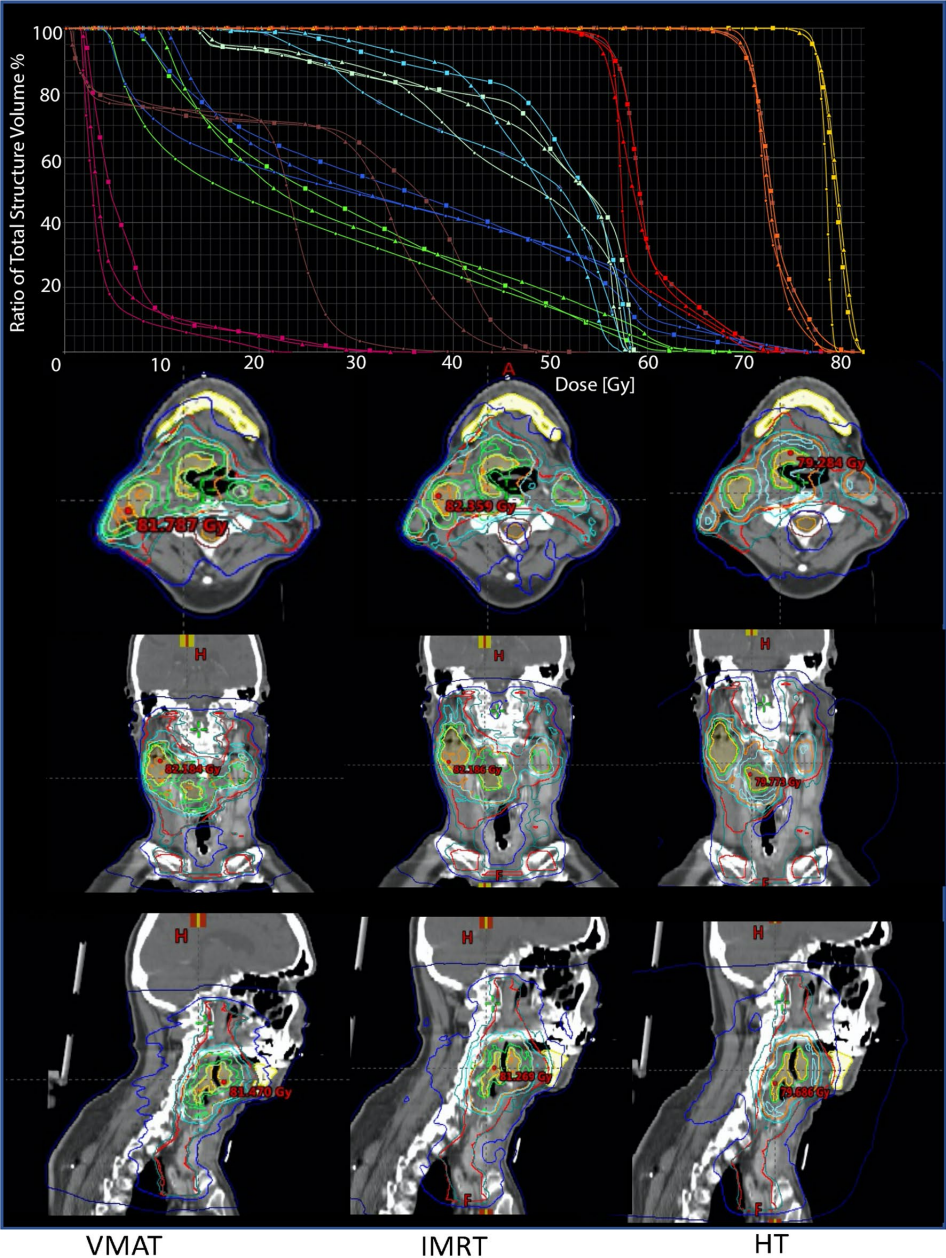
Examples of isodose distribution and DVH of VMAT, IMRT and HT for patient 2. SIB colours: SIB 77 Gy—yellow, SIB 70 Gy—orange, SIB 56 Gy—red. OAR colours: brainstem—magenta, left parotid gland—green, right parotid gland—blue, spinal cord—brown, left plexus brachialis—light green, right plexus brachialis—light blue

For SIB56Gy, the coverage parameter CVF was comparable in all three RT technique plans between 0.983 ± 0.006 and 0.986 ± 0.004. In contrast to the SIB77Gy and SIB70Gy, the SIB56Gy conformity analysis using HTCI and CN favors VMAT (HTCI: 0.747 ± 0.024 and CN 0.739 ± 0.023). IMRT (HTCI: 0.731 ± 0.029; CN: 0.723 ± 0.031) and HT (HTCI: 0.728 ± 0.034; CN: 0.719 ± 0.033) achieved lower results. Plan examples are given in Fig. 1.

The comparison check done by Student’s two-sided paired t-test (IMRT vs. HT, IMRT vs. VMAT, and HT vs. VMAT) reveals a significant difference concerning D_mean_, D_2%_ and D_5%_ for SIB77Gy between all RT techniques, favoring HT. D_5%_ differed significantly for SIB70Gy and SIB56Gy with the best results by HT.

D_mean_ to the ipsilateral parotid gland varied between 31.1 ± 4.0 Gy (HT), 33.1 ± 4.4 Gy (IMRT) and 32.8 ± 4.8 Gy (VMAT) and contralateral between 22.6 ± 5.5 Gy (HT), 26.2 ± 3.6 Gy (IMRT) and 26.3 ± 3.9 Gy (VMAT). HT showed minimally better results for ipsilateral parotid glands vs. VMAT and IMRT. IMRT and VMAT achieved comparable results. For the contralateral parotid gland, there was only an advantage of HT compared to VMAT (p = 0.043).

The D_max_ for the spinal cord plus 5 mm was comparably safe for all kinds of radiotherapy. Interestingly, HT reached the lowest D_max_ 45.7 ± 8.2 Gy, but the highest standard deviation.

For comparison of the plexus brachialis, this OAR was also discriminated between ipsilateral and contralateral localization. Concerning D_2%_ only for the contralateral plexus, a small significant difference was reached for IMRT vs. VMAT, favoring IMRT.

The D_mean_ of the mandible was 44.2 ± 8.5 Gy for HT, 46.0 ± 7.9 Gy for VMAT, and 48.7 ± 6.7 Gy for IMRT. VMAT and HT were significantly better than IMRT (VMAT vs. IMRT p = 0.025). D_mean_ of the mandible could be lowered by HT compared to VMAT (p = 0.014). D_2%_ for mandible was comparably high for all used techniques (VMAT: 70.7 ± 3.6 Gy; IMRT: 71.2 ± 2.8 Gy and HT: 71.9 ± 2.7 Gy).

## Discussion

The aim of this planning study was to investigate the planning options for the first six patients enrolled in the pre-study of the ESCALOX trial. Thus, IMRT, VMAT, and HT planning algorithms and the corresponding RT plans were compared and checked for the constraints as determined by the trial protocol [12]. Different plan quality parameters were calculated from DVH for all planning target volumes (SIB77Gy, SIB70Gy, and SIB56Gy) in order to compare all RT techniques. Dose coverage, dose conformity, and homogeneity were analyzed. For SIB77Gy, SIB70Gy, and SIB56Gy, the coverage of all RT modalities was comparable. HT gained best results for SIB77Gy concerning conformity (HTCI and CN) and homogeneity defined by the HI. Concerning coverage (CVF) of HT for SIB77Gy, there was a small gap between HT and VMAT and IMRT, which seems clinically irrelevant.

The SIB70Gy volume encompassed the shell between the margin of SIB77Gy and SIB70Gy. Best coverage for SIB70Gy was achieved by IMRT. Conformity and homogeneity favored HT. The analysis of SIB56Gy—the volume for elective nodal irradiation—revealed a comparable coverage for all RT modalities. Best conformity was reached by VMAT, superior homogeneity by HT. Thus, HT is favored for conformity defined by HTCI and CN for the SIB77Gy and SIB70Gy. Best performance for homogeneity (HI) in all SIBs was achieved only by HT, followed by IMRT. Important DVH parameters describing the steepness of the dose gradient as D_2_ or D_5_ as well as D_mean_ showed better results for HT, but no differences between IMRT and VMAT. Despite the shown advantage of HT, all RT modalities fulfilled the dose specification of the trial protocol: 50% of the PTV volumes (SIB77, SIB70, SIB56) should receive no more than 79.3 Gy, 72.1 Gy, and 57.7 Gy, respectively.

Differding et al. compared in a RT planning study the potential of VMAT and HT in part 1 for best target coverage and in part 2 for maximal OAR sparing [5, 21]. This was an FDG-PET based delineation for dose painting planning study on datasets of five patients with oropharyngeal cancer. HT and VMAT achieved comparable results for target coverage (PTV 70 Gy (SIB) and PTV 56 Gy applied in 35 fractions) and OAR sparing. Slight differences were seen for conformity of PTV 56 Gy favoring HT. The authors concluded that HT and VMAT were able to deliver similar dose distributions for FDG-PET-based dose escalation for the concept of dose painting by numbers. The good results for VMAT were at the cost of four arcs. The planning physicists extended the limits of the TPS by re-optimization of the RT plans after comparing their results to the other TPS results. For our planning scenario, both physicists did the same. In order to yield comparable results for target coverage and to minimize the OAR dose, VMAT applies the dose by using three arcs. Despite one additional arc compared to a routine head and neck RT plan, the beam on time is lower than the amount of time of application of an HT plan. This indicates that the probability of intra-fraction motion is smaller by VMAT than with HT.

Thorwarth et al. compared 9-field step and shoot IMRT with HT for inhomogeneous dose distribution by dose painting by numbers for dose escalation to hypoxic subvolumes. HT achieved a higher degree of conformity. Therefore, in this planning competition, the authors summarized both implemented techniques (IMRT and HT) as suitable for dose escalation [6]. Only small differences for target coverage were seen by Stromberger et al. for planning comparison of a SIB concept in bilateral and unilateral neck irradiation for intensity modulated proton therapy (IMPT), HT, and VMAT [3].

Concerning OARs, the comparison of DVH parameters for parotid gland, brainstem, and mandible saw HT ahead in our investigation. Despite the favored position of HT in many parameters, IMRT and VMAT ensured safe coverage of target volumes and adherence to the OAR constraints per trial protocol. The small differences seen in the head-to-head comparison will probably have no impact on clinical outcome. Other groups investigating different RT techniques and fractionation concepts for head and neck cancer hypothesized no clinical impact of small dosimetric differences [5, 7] as well. Comparing D_mean_ to the ipsilateral parotid gland, we achieved the lowest dose by HT (31.1 Gy). There was a difference of 2 Gy compared to IMRT (33.1 Gy). HT reached also best contralateral D_mean_ (22.6 Gy) to the parotid gland (VMAT and IMRT > 26 Gy). Jacob et al. investigated the influence of leaf and jaw parameter on plan quality for head and neck RT in nine patients by IMRT, VMAT, and HT. They concluded that all plans fulfilled OAR constraints, but HT lowered the dose to the parotid glands by best dose homogeneity. Changes of leaf thickness and jaw width had no additional benefit for OAR sparing [22]. Inconsistent results favoring one technique over another for dose reduction to salivary glands are reported [9, 23–27]. The groups of Wiezorek [27] and Holt [28] examined head and neck RT plans created by diverse IMRT techniques and various machine parameters between different institutions, the conclusion was the same as in many single center experiences: good target coverage and acceptable OAR sparing for all techniques with slight preference. At one point, HT was superior, on occasion VMAT or IMRT. Both multicenter planning comparison trials delineated the PTV (respective SIB volumes) and OARs in the core unit. Thus, the uncertainty in target volume delineation was excluded and the results clearly concentrate on the physical aspect of RT planning and treatment delivery. The aim of this study consisted in proof of principle for using different TPS for planning of real patients in preparation of a multicenter study. For the purpose of initiating a multicenter IMRT-trial, a dummy run for all participating centers with a central QA core unit would be necessary. Early on, this was recommended by Wallner et al. in 1989 [29] and renewed by McDowell in 2019 [10]. In order to reduce unnecessary dose deposition to OARs and surrounding healthy tissue, the trial protocol recommended triple re-planning [12] based on results of the work of Duma et al. [30] and others e.g. [31]. The onboard imaging units of linear accelerators increase the accuracy of patient positioning (daily CB-CT, MV-CT) and allow for adaptive planning. Consequently, the target volumes and OARs can be observed and adapted if necessary.

This analysis was done with the primary image set for initial RT planning. The re-planning scenarios as performed for each patient during the pre-study are not shown.

MRI for GTV definition is important for safety of target and OAR delineation due to better spatial resolution. Over the last years, many results were confirmed for implementation of molecular imaging and widened sequences of MRI, e.g. diffusion-weighted MRI for GTV definition [32] and also better definition of OAR, for instance, swallowing structures [33]. In a dummy run, Felice et al. demonstrated the smaller GTV definition and lower inter-observer variability for MRI-based contouring compared to CT-based target volume definition [34]. Implementation of molecular imaging is a widely investigated field, not only in RT planning of head and neck cancer [35]. Some trials investigating dose escalation integrate the PET signal by employing different tracers and voxel-based RT-planning [5, 6, 36–39]. Hence, the dose escalation strategy based on PET data to overcome troubleshooters like hypoxia or proliferation is not clear [40]. Until now, no large randomized trial on this topic has been published. Despite the advent of implementation of biological imaging into target volume delineation for head and neck cancer treatment aimed at attacking, for instance, hypoxic subvolumes by dose escalation, we resigned at the level of the pre-study of the performance of 18-F-FMISO-PET.

We generated in one center the presented results by calculating 18 different RT plans for a dose escalation study by using the two TPS on datasets of six real patients. The planning group consisted of two radiation oncologists (SP and MD) and two physicists (SK and MO) experienced in the TPS for head and neck cancer planning. Our results are comparable to those of other investigators of single or multicenter planning studies despite our homogeneous in-house team. The statistical results of small sample sizes always require careful interpretation. A significant difference detection calls for a defined number of dose values or a large difference between the values of the comparators. Possible sources for bias are discussed by van Gestel et al. contributing to different interpretations of planning comparisons by various investigators. One of the drawbacks of our study is the use of only those TPS which were available at our department and the limited number of patients. However, in light of these limitations, it was possible to reach the planning objectives defined by the ESCALOX trial protocol [12].

## Conclusion

The analysis of the quality parameters for RT planning revealed that the tested TPS can produce comparable plan quality for this dose escalation trial. Despite the very good results of HT planning for coverage, conformity, and homogeneity of these plans, also the TPS for calculation of VMAT and IMRT achieved acceptable results for a double simultaneous integrated boost concept for dose escalation in head and neck cancer delivered by IMRT or VMAT. The tested TPS and IMRT-techniques are allowed for the ESCALOX trial.

## Supplementary information

**Additional file 1.** Raw data from DVH for analysis of plan comparison parameter.

## Data Availability

The dataset supporting the conclusions of this article is included within the article and its Additional file 1.

## Abbreviations

AAA: Anisotropic Analytical Algorithm
ALARA: As Low as Reasonable Achievable
CB-CT: Cone beam CT
CN: Conformation number
COIN: Conformity index
CT: Computed tomography
CVF: Coverage factor
D: Dose
DVH: Dose Volume Histogram
FDG-PET: Fluorodesoxyglucose positron emission tomography
18-F-FMISO-PET: 18-Fluoro-Fluoromisonidazol positron emission tomography
GTV: Gross tumor volume
HI: Homogeneity index
HT: Helical tomotherapy
HTCI: Healthy tissue conformity index
IMRT: Intensity Modulated RadioTherapy
MRI: Magnetic Resonance Imaging
MV-CT: Megavoltage CT
OAR: Organ at risk
PTV: Planning target volume
QA: Quality assurance
RT: Radiotherapy
SIB: Simultaneous integrated boost
TPS: Treatment planning system
TV: Target volume
TVRI: Irradiated target volume encompassed by reference dose 95%-isodose
VMAT: Columetric Modulated Arc Therapy
VRI: Irradiated volume encompassed by reference 95%-isodose
